# Correlation of Brain Metastasis Shrinking and Deviation During 10-Fraction Stereotactic Radiosurgery With Late Sequela: Suggesting Dose Ramification Between Tumor Eradication and Symptomatic Radionecrosis

**DOI:** 10.7759/cureus.33411

**Published:** 2023-01-05

**Authors:** Kazuhiro Ohtakara, Kuniaki Tanahashi, Takeshi Kamomae, Kazushi Miyata, Kojiro Suzuki

**Affiliations:** 1 Department of Radiation Oncology, Kainan Hospital Aichi Prefectural Welfare Federation of Agricultural Cooperatives, Yatomi, JPN; 2 Department of Radiology, Aichi Medical University, Nagakute, JPN; 3 Department of Neurosurgery, Gifu Prefectural Tajimi Hospital, Tajimi, JPN; 4 Department of Neurosurgery, Nagoya University Graduate School of Medicine, Nagoya, JPN; 5 Department of Radiology, Nagoya University Graduate School of Medicine, Nagoya, JPN; 6 Department of Surgical Oncology, Nagoya University Graduate School of Medicine, Nagoya, JPN

**Keywords:** internal margin, inter-fractional change, image guidance, large tumor, frameless, fractionation, brainstem, brain tolerance, brain radionecrosis, brain metastasis

## Abstract

Stereotactic radiosurgery (SRS) with >5 fraction (fr) has been increasingly adopted for brain metastases (BMs), given the current awareness of limited brain tolerance for ≤5 fr. The target volume/configuration change and/or deviation within the cranium during fractionated SRS can be unpredictable and critical uncertainties affecting treatment accuracy, plus the effect of these events on the long-term outcome remains uncertain. Herein, we describe a case of two challenging BMs treated by 10 fr SRS with a unique dose-gradient optimization strategy, in which the large cystic tumor revealed an intriguing correlation of such inter-fractional change with late radiographic sequela, suggesting a dose threshold for attaining long-term local tumor control and being immune to symptomatic brain necrosis.

A 63-year-old man presented with two cystic lesions located in the left parietal lobe (19.9 cm^3^) and pons (1.1 cm^3^) one month after surgery for esophageal squamous cell carcinoma. The principles for 10 fr SRS were as follows: (1) very inhomogeneous gross tumor volume (GTV) dose covered by 53 Gy, biologically effective dose with an alpha/beta ratio of 10 (BED_10_) of ≥80 Gy; (2) moderate dose spillage margin outside the GTV boundary: 2-2.5 mm outside the GTV margin was covered by 37 Gy, BED_10_ of ≈50 Gy; (3) concentrically-laminated, steep dose increase inside the GTV boundary: 2 mm inside the GTV margin was covered by ≥62 Gy, BED_10_ of ≥100 Gy. At the completion of SRS, the parietal lesion showed significant shrinking and dorsomedial shifting with slight evisceration of the GTV, followed by marked regression of the parietal lesion within four months. At 13.5 months, a cystic change was noted at the dorsal part of the remnant. At 16.7 months, ventral enhancement gradually expanded without enlargement of the dorsal cystic component. On the T2-weighted images, the dorsal low-intensity remnant and ventral iso-intensity blurry-demarcated component were contrasting. Pathological examinations during and after lesionectomy at 17.4 months revealed necrosis only. At 30.5 months, the patient had a left visual field defect without recurrence. In contrast, the pons lesion showed no notable change during 10 fr SRS and nearly complete remission over six months with its sustainment without radiation injury at 30.5 months.

Taken together, 10 fr SRS with a sufficient BED_10_ can provide superior tumor response and safety for BM that is not amenable to ≤5 fr SRS. Although a very inhomogeneous GTV dose can contribute to early and adequate tumor shrinkage and subsequent local tumor eradication, significant tumor shrinkage during fractionated SRS (fSRS) inevitably results in unnecessary higher dose exposure to the surrounding brain, which could lead to late radiation injury requiring intervention. The optimum dose should be determined through further investigation, in consideration of the dynamic and unpredictable nature of the actual absorbed doses to both the tumor and the surrounding brain.

## Introduction

With the advent and development of efficacious anti-cancer medication penetrating brain metastases (BM), stereotactic radiosurgery (SRS), either single- or multi-fraction, with conservation of whole brain radiotherapy (WBRT) as a last resort, has increasingly become significant and prevalent in the management of BM, to prevent both acute and late detrimental effects relevant to WBRT and to preserve better neurocognitive function [1,2]. Furthermore, both long-term excellent local control and safety have been strongly anticipated for SRS in substantial number of BM cases as a result of improved prognosis and prolonged survival [3]. SRS is also expected for challenging BM cases, such as >3 cm, as a far less invasive alternative to open surgery, which entails the risk of provoking cerebrospinal tumor seeding besides the inherent invasiveness and burden on patients.

Fractionated SRS (fSRS) with more than 5 fractions (fr) has been increasingly adopted for BM, especially with large volumes and/or eloquent locations to enhance its efficacy and safety, as there is contemporary awareness of far less limited brain tolerance for ≤5 fr SRS than previously assumed [3-5]. However, the optimum fractionation number, dose distribution, brain tolerance, and indication for SRS of >5 fr, for example, 10 fr, remain unclear. In addition, the most appropriate model of the biological effective dose (BED) that can convert any fractionated dose to a single-fraction equivalent dose, remains controversial [3]. Moreover, the target volume/configuration change and/or deviation within the cranium during fSRS, especially over one week, can be unpredictable and cause critical uncertainty affecting treatment accuracy, as the currently prevailing image guidance has been generally based on bone matching, not the tumor itself [6-8]. Such events, if noticeable, can inevitably lead to a gradual discrepancy between the planned and delivered doses to both the tumor and the surrounding normal tissue [7,8]. Since the clinical introduction of frameless fSRS with ≥5 fr for BM at the first author’s institution in 2009 [6], these possible uncertainties have been considered and observed in some cases [7]. Thus, this cautionary report was published for the first time in 2014 [7]. These possible tumor changes during the period from the planning image acquisition to the treatment completion, along with its variable patterns (shrinking or enlarging) and the inextricable difficulty in prediction have recently been acknowledged by several investigators [9-14]. However, the effect of these events on the long-term outcome remains uncertain and needs to be elucidated to further optimize the dose distribution for fSRS, specifically the degree of dose gradient both inside and outside the tumor boundary and the tumor dose inhomogeneity [15-17].

Herein, we describe a case of two BMs deemed not amenable to ≤5 fr SRS, which were treated with 10 fr SRS. In this case, the large cystic lobar lesion revealed an intriguing correlation between remarkable inter-fractional change and late radiographic sequelae associated with aggravation of relevant neurological symptoms. In contrast, the other brainstem lesion showed no significant change during fSRT and nearly complete remission within approximately six months with sustainment without radiation injury at 30.5 months. These two contrasting clinical courses suggest the dose ramifications for attaining complete local tumor eradication while being immune to symptomatic brain necrosis.

The synopsis of this study was previously presented at the 35th Annual Meeting of the Japanese Society for Radiation Oncology held on November 10-12, 2022.

## Case presentation

**Figure 1:**
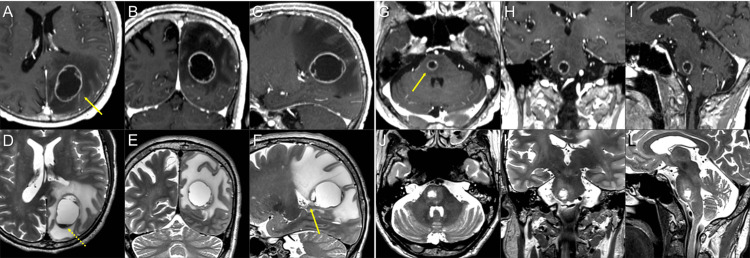
Magnetic resonance images for 10-fraction stereotactic radiosurgery planning. The images show contrast-enhanced (CE) T1-weighted images (T1-WI) (A-C, G-I); T2-weighted images (T2-WI) (D-F, J-L); axial images (A, D, G, J); coronal images (B, E, H, K); and sagittal images (C, F, I, L). (A-F) A cystic lesion (arrow in A) in the left parietal lobe is associated with massive surrounding edema and fluid sedimentation within the cyst (dashed arrow in D). The ventral side of the lesion is near the lateral ventricular wall (arrow in F). (G-I, J-L) A cystic lesion (arrow in G) in the pons with mild perilesional edema.

A 63-year-old man presented with unsteadiness and right-sided hemiparesis. One month prior, the patient had undergone definitive surgery (video-assisted thoracoscopic surgery), following three courses of neoadjuvant chemotherapy consisting of docetaxel, cisplatin, and 5-fluorouracil (DCF) for squamous cell carcinoma (SCC) of the lower thoracic esophagus. The initial clinical and postoperative pathological stages were III (cT3 N2 M0) and IIIB (ypT2 N2 M0), respectively, based on the tumor, node, and metastasis (TNM) grading system defined by the Eight Union for International Cancer Control criteria. ^18^F-fluorodeoxyglucose positron emission tomography/computed tomography obtained two days before surgery showed a partial response of the preexisting thoracic lesions and any obvious abnormalities in the brain, even if reviewed retrospectively. The patient initially noticed right-sided weakness 36 days after the surgery and then experienced sensory impairment, visual field defect, dysarthria, and unsteadiness in gait with gradual worsening over a few days. Imaging studies showed two cystic-enhancing lesions located in the left parietal lobe and pons, with a maximum diameter of 41 mm and 16 mm, respectively (Figures 1A-1L).

**Figure 2:**
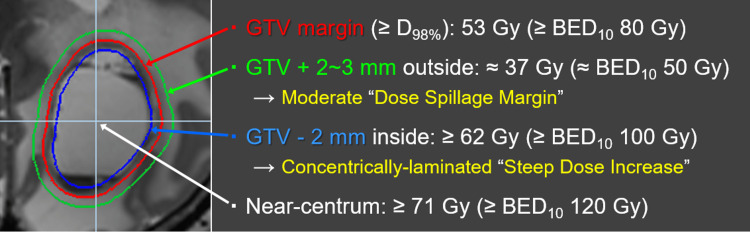
Principles of dose prescription and distribution design for 10-fraction stereotactic radiosurgery of brain metastases. The most prioritized foundation for dose distribution design is the gross tumor volume (GTV) boundary, not the margin-added planning target volume (PTV). The dose prescription is based on the biologically effective dose (BED) derived from the linear-quadratic formula with an alpha/beta ratio of 10 (BED_10_) and three-tiered dose gradient optimization both just outside and inside the GTV boundary. The GTV margin was covered by 53 Gy, with a BED_10_ of ≥80 Gy, which was highly prioritized. To ensure a moderate, not too steep or gradual, dose spillage (attenuation) margin outside the GTV boundary, 2-3 mm outside the GTV margin was covered by 37 Gy, with a BED_10_ of ≈50 Gy. To attain a concentrically laminated steep dose increase inside the GTV boundary, 2 mm inside the GTV margin was covered by ≥62 Gy, with a BED_10_ of ≥100 Gy, by intentionally increasing the centrum dose by ≥71 Gy, a BED_10_ of ≥120 Gy. The BED_10_ values of 37, 53, 62, and 71 Gy correspond to 50.7, 81.1, 100.4, and 121.4 Gy, respectively. The constancy of the maximum dose or % isodose of 53 Gy, that is, the degree of GTV dose inhomogeneity, is considered insignificant, with the variations being allowed up to approximately 106 Gy at the maximum dose.

**Figure 3:**
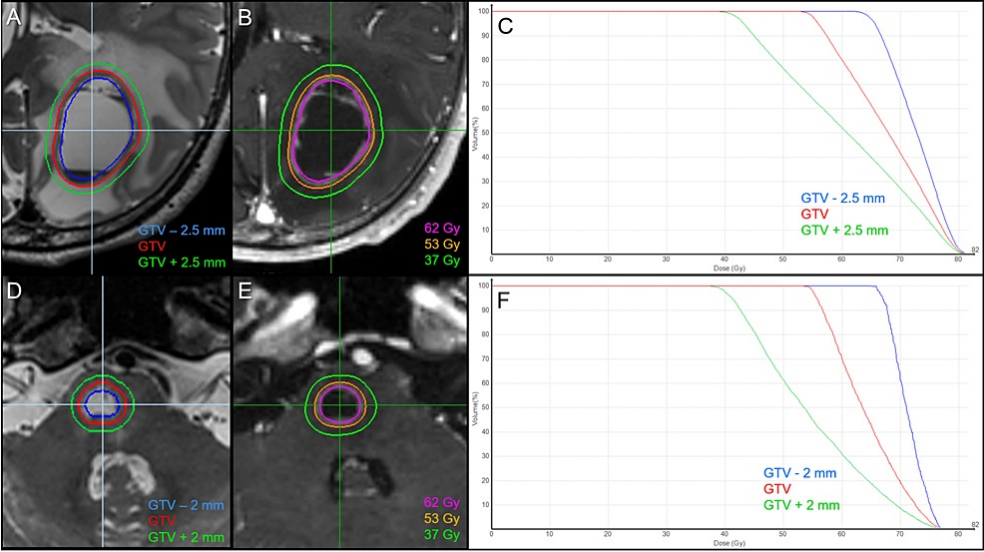
Target definitions, planned dose distributions, and dose-volume histograms. The images show the parietal lesion (A-C); the pons lesion (D-F); target definitions including gross tumor volume (GTV) and two other objects for dose evaluation (A, D); planned dose distributions (B, E); and dose-volume histograms (C, F).

These neurological symptoms were attributed mainly to the mass effect of the parietal lesion associated with massive perilesional edema. Through multi-disciplinary deliberation, these BMs were initially treated with 10 fr of SRS (Figures 2, 3A-3F, and Table 1). Each gross tumor volume (GTV), 19.93 cm^3^ and 1.09 cm^3^, was defined as encompassing the contrast-enhancing lesion that was almost consistent with the visible mass on T2-weighted images (T2-WI) (Figures 1A-1L, 3A-3F).

**Table 1:** Defined target volumes and relevant dosimetric parameters for planned dose distributions. The dose calculation algorithm is ray-tracing with a calculation grid size of 0.98x1.00x0.98 mm. The maximum doses of the gross tumor volume (GTV) in the parietal lobe and pons are 81.6 and 76.9 Gy, respectively. The percentage of isodose was normalized to 100% at each maximum dose. Equivalent dose in 2 Gy fractions (EQD2) is the biologically effective dose with an alpha/beta ratio of 10 (BED_10_), equivalent total dose delivered at 2 Gy per fraction.

Location	Target for evaluation	Target volume	Coverage	Isodose (%)	Absolute dose	BED_10_	EQD2
Parietal lobe	GTV +2.5 mm	30.67 cm^3^	100%	45.3%	37 Gy	50.7 Gy	42 Gy
			98%	51.1%	41.7 Gy	59.1 Gy	49 Gy
	GTV	19.93 cm^3^	99.8%	65.0%	53 Gy	81.1 Gy	68 Gy
			98%	67.3%	54.9 Gy	85.0 Gy	71 Gy
	GTV -2.5 mm	11.97 cm^3^	99.9%	76.0%	62 Gy	100.4 Gy	84 Gy
			98%	78.9%	64.4 Gy	105.9 Gy	88 Gy
Pons	GTV +2 mm	2.46 cm^3^	100%	48.1%	37 Gy	50.7 Gy	42 Gy
			98%	51.8%	39.8 Gy	55.6 Gy	46 Gy
	GTV	1.09 cm^3^	99.8%	68.9%	53 Gy	81.1 Gy	68 Gy
			98%	71.5%	55.0 Gy	85.3 Gy	71 Gy
	GTV -2 mm	0.33 cm^3^	99.9%	80.6%	62 Gy	100.4 Gy	84 Gy
			98%	86.1%	66.2 Gy	110.0 Gy	92 Gy

fSRS was implemented using CyberKnife (CK) M6® (Sunnyvale, CA: Accuray Inc.) with a dedicated treatment planning system (TPS) Precision® (Sunnyvale, CA: Accuray Inc.). Instead of separate planning for each tumor, simultaneous and comprehensive optimization with a single plan (path) was adopted to efficiently irradiate multiple targets with 6 MV x-rays, 127 beams from 71 nodes, for which the dedicated regular dodecagon-shaped variable-sized collimator, namely Iris® (Sunnyvale, CA: Accuray Inc.), was used with collimator sizes of 10, 15, 20, 25, 30, and 35 mm. The optimization algorithm was the latest CK-VOLO® (Sunnyvale, CA: Accuray Inc.). The estimated treatment time (ETM) calculated on the TPS was 28 minutes per fraction. This comprehensive sequential multi-target irradiation technique with a single-path plan was inimitably established to significantly reduce treatment time while retaining the plan's qualities to expand the application of CK for multiple BM. In 27 cases with five to 12 BM, the mean ETM was 40.2 minutes per fraction (6.0 minutes per lesion) with a maximum of 52 minutes.

Since 2018 we have implemented our principles of dose prescription and distribution design to attain superior tumor response and its long-term sustainment with higher rates, irrespective of differences in treatment modalities or techniques. These are summarized as follows (Figure 2): (1) In typical cases where the configuration of the enhancing mass is equal to or slightly larger than the visible mass on T2-WI, the GTV is defined as an enhancing lesion, as mentioned above (Figures 1A-1L, 3A-3F). Thus, T1/T2 matching (contrast-enhanced T1-weighted image {CE T1-WI} vs. T2-WI) is mandatory. In cases with T1/T2 mismatch, such as excessive exudation of contrast media or exiguous enhancement, a visible mass on T2-WI is prioritized. (2) Preservation of the BED to the GTV margin, irrespective of tumor volume and/or location, along with optimization of the dose gradient both just inside and outside the GTV boundary. We use BED-based three-tiered dose prescription near the GTV boundary and not the single isodose prescription for the margin-added planning target volume (PTV) margin as usual. The BED is based on a linear-quadratic formula with an alpha/beta ratio of 10 (BED_10_), and a GTV margin of ≥98% (D_98%_) is covered by a BED_10_ of ≥80 Gy with highest priority. The dose gradient optimization strategies just outside and inside the GTV boundary are described in Figure 2. (3) To minimize the risk of radiation injury, various flexible dose fr (3-15 fr, mainly 3, 5, 8, and 10 fr) were subtilized according to the GTV size, location, and the distances between multiple targets to reduce the susceptibility to dose interference.

**Figure 4:**
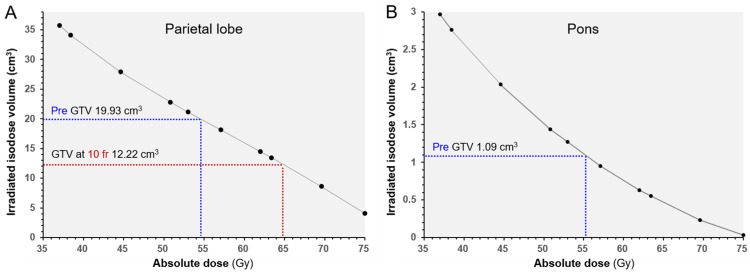
Irradiated isodose volumes compared to each gross tumor volumes before stereotactic radiosurgery and at 10 fractions. The images show (A) the parietal lesion and (B) the pons lesion. The irradiated isodose volumes (IIV) are the total volumes, including the gross tumor volume (GTV) and surrounding brain, receiving at least the corresponding doses. The dashed lines show the IIV and dose corresponding to each GTV.

**Figure 5:**
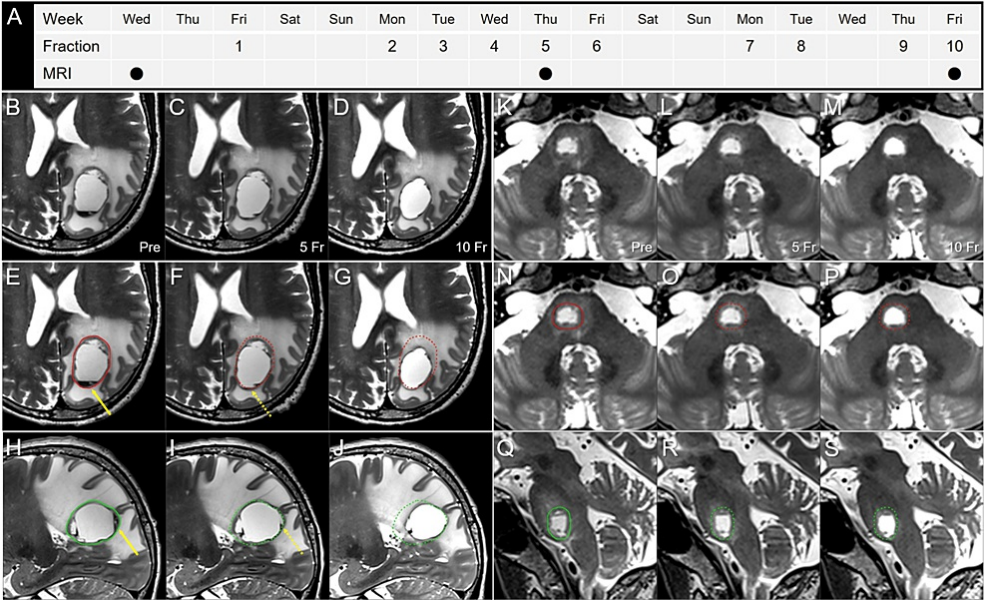
T2-weighted images obtained two days before stereotactic radiosurgery, at 5 and 10 fractions. The images show (A) the actual schedule for fractionated SRS (fSRS) and MRI acquisition; (B-J) the parietal lesion; (K-S) the pons lesion; (B-G, K-P) axial images; (H-J, Q-S) sagittal images; (B, E, H, K, N, Q) before fSRS (pre); (C, F, I, L, O, R) at 5 fraction (fr); and (D, G, J, M, P, S) at 10 fr. These T2-weighted image datasets were co-registered to the dedicated workstation, MIM Maestro^TM^ (Cleveland, OH: MIM Software) and then fused to coincide with each other through the cranium. The pre-fSRS GTVs were contoured in red and green for the axial and sagittal images, respectively. In the parietal lesion, slight dorsomedial displacement of the lesion along with disappearance of the preexisting fluid sedimentation (arrows in E, H) was observed at 5 fr (dashed arrows in F, I). At 10 fr, a 39% (7.71 cm^3^) reduction in the GTV (19.93 to 12.22 cm^3^) and dorsomedial shifting of the tumor center were noted along with an improvement in the mass effect. Slight evisceration of the GTV dorsomedially from the initial dimensions was found ad eundem at 5 and 10 fr. In the pons lesion, no significant change was observed, apart from regression of the mild surrounding edema.

The actual planned dose distributions and relevant parameters are shown in Figures 3A-3F, 4A, 4B, and Table 1. fSRS commenced two days after the planning image acquisition, and T2-WI was also obtained at 5 and 10 fr for evaluation of any inter-fractional change (Figures 5A-5S).

In the parietal lesion, slight evisceration of the GTV dorsomedially from the initial GTV dimensions and disappearance of fluid sedimentation level in the cyst content were observed at 5 fr, although fSRT continued as per the initial plan. At the completion of 10 fr, however, significant GTV shrinking (61% of the initial volume) and the same degree of dorsomedial GTV evisceration were observed, although a significant deviation of the lesion center within the cranium was noted. These inter-fractional changes resulted in an unexpected higher dose exposure to the ventral paraventricular white matter. The neurological symptoms gradually improved during fSRS and fully resolved within almost one month.

Nivolumab (240 mg per body) was administered six days after the fSRS completion, however, further anti-cancer medication had to be jettisoned due to symptomatic lung adverse reaction developing two weeks after the initiation, for which steroid administration with gradual tapering-off was required over three months.

**Figure 6:**
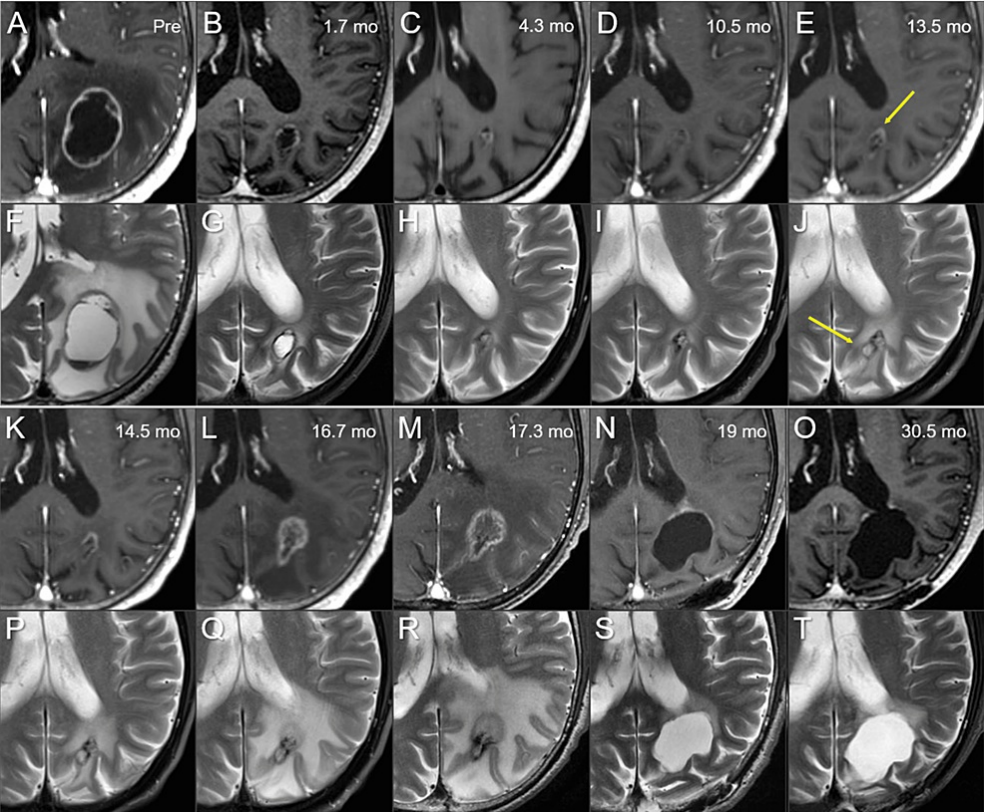
Serial magnetic resonance images for brain metastasis in the left parietal lobe. The images show (A-E, K-O) axial CE-T1-WI; (F-J, P-T) axial T2-WI; (A, F) before fSRS (pre); (B, G) at 1.7 months (mo); (C, H) at 4.3 months; (D, I) at 10.5 months; (E, J) at 13.5 months; (K, P) at 14.5 months; (L, Q) at 16.7 months; (M, R) at 17.3 months; (N, S) at 19 months; (O, T) at 30.5 months. These serial image datasets were co-registered and fused on MIM Maestro^TM^ (Cleveland, OH: MIM Software). The volumes of the visible mass on CE-T1-WI and/or T2-WI at 4.3 and 17.3 months are 0.94 and 3.19 cm^3^, respectively. At 13.5 months, increased enhancement (arrow in E) and a cystic change (arrow in J) are noted at the ventral and dorsal part of the remnant lesion, respectively. CE: contrast-enhanced; T1-WI: T1-weighted image; T2-WI: T2-weighted image

The parietal lesion showed marked regression of both the tumor and the surrounding edema, leaving a low-intensity scar-like lesion on T2-WI (0.94 cm^3^, 4.8% of the initial GTV), with exiguous enhancement at 4.3 months (Figures 6A-6T).

**Figure 7:**
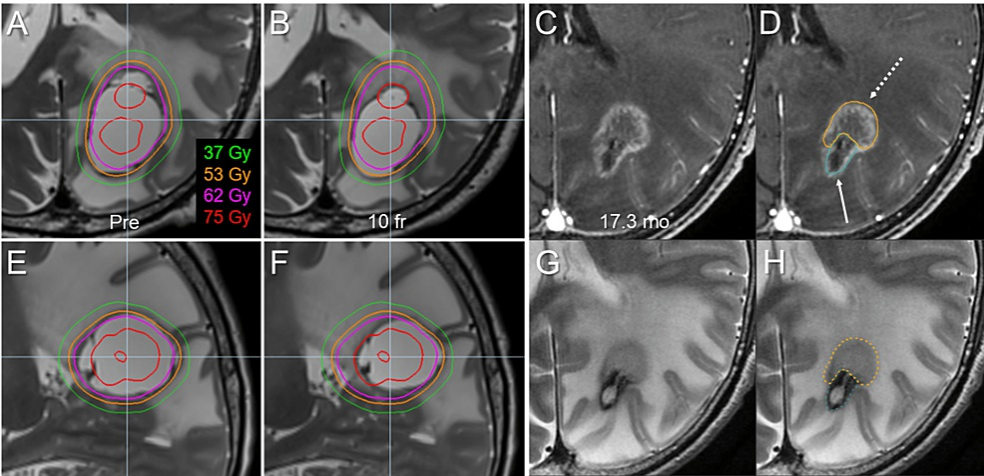
Discrepancy between planned and actually absorbed doses at the completion of stereotactic radiosurgery and its comparison with magnetic resonance images before lesionectomy. Axial images (A-D, G, H); sagittal images (E, F); T2-WI (A, B, E-H); CE-T1-WI (C, D); before fSRS (pre) (A, E); at 10 fr (B, F); at 17.3 months after fSRS, just before craniotomy (C, D, G, H). Planned representative isodose lines are superimposed onto T2-WI (A, B, E, F). Of note, the maximum dose to the adjacent brain parenchyma finally increased up to 7.5 Gy per fraction at the ventral side of the GTV (B, F). On T2-WI, the dorsal low-intensity stable remnant (highlighted in light blue, arrow in D) and the ventral iso-intensity blurry-demarcated enlarging component (orange, dashed arrow in D) are contrasting. CE: contrast-enhanced; T1-WI: T1-weighted image; T2-WI: T2-weighted image

Although the marked regression was sustained at 10.5 months, a slight increase in enhancement at the ventral part of the remnant, along with cystic enlargement at the dorsal part, was found concomitantly with increased perilesional edema at 13.5 months, suggesting potential tumor regrowth from the under-coverage part with 53 Gy. At 16.7 months, the ventral enhancement gradually expanded with aggravation of the perilesional edema without enlargement of the dorsal cystic component, while the patient presented with no neurological deterioration. At 17.2 months, however, the patient experienced seizures with subsequent neurological worsening, including right-sided weakness, speech impairment, and visual field defects. Magnetic resonance images (MRI) at 17.3 months showed further enlargement of the ventral enhancing component with transformation into a feathery pattern without enlargement of the dorsal cystic component. Of note, the dorsal low-intensity remnant and ventral iso-intensity blurry-demarcated component were contrasting on T2-WI. These findings were significantly correlated with the dynamic dosimetric changes in the surrounding brain, as well as the GTV during fSRS (Figures 7A-7H).

The patient underwent lesionectomy at 17.4 months via The ViewSite^TM^ Brain Access System (VBAS®) (Boca Raton, FL: Vycor Medical). During surgery, an elastic hard mass suggestive of necrosis, containing a liquidized degenerated tissue contiguous to the lateral ventricular wall, was extirpated in its entirety. Both intraoperative and permanent pathological examinations revealed necrosis along with reactive changes in the brain parenchyma without any malignancy. The patient gradually recovered leading an independent life despite experiencing visual field disturbances and infrequently experiencing seizures. At 23.2 months, MRI revealed slight enlargement of the ventral enhancement near the cavity, without subsequent aggravation at 30.5 months (Figures 6A-6T).

**Figure 8:**
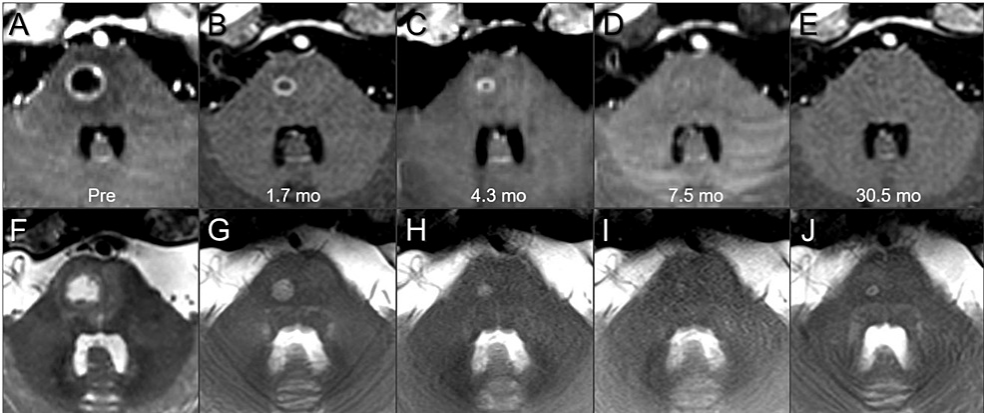
Serial magnetic resonance images for brain metastasis in the pons. The images show (A-E) axial CE-T1-WI; axial T2-WI (F-J); before fSRS (pre) (A, F); at 1.7 months (mo) after fSRS (B, G); at 4.3 months (C, H); at 7.5 months (D, I); and at 30.5 months (E, J). These serial image datasets were co-registered and fused on MIM Maestro^TM^ (Cleveland, OH: MIM Software). CE: contrast-enhanced; T1-WI: T1-weighted image; T2-WI: T2-weighted image

In contrast, the pons lesion showed no notable change during fSRS (Figures 5A-5S), gradual tumor shrinkage over several months, and nearly complete remission at 7.5 months with its sustainment at 30.5 months (Figures 8A-8J). At 30.5 months, the patient’s general condition was unremarkable, except for a visual field defect, with no evidence of recurrence.

## Discussion

In cases with isolated central nervous system (CNS) metastases without extra CNS active disease, especially limited BM, long-term local tumor control and safety have been unprecedentedly required for SRS, as is the presented case. In such cases, when the maximum response following SRS results in a partial response with residual viable tissue, the BM would eventually and inevitably represent regrowth unless continuous anti-cancer medication efficaciously inhibits this [2]. Furthermore, in symptomatic cases, early and sufficient BM shrinking without aggravation of the surrounding parenchymal edema and elimination of the relevant mass effect and symptoms are desirable, irrespective of the expected prognosis. To attain initial excellent tumor response and its long-term sustainment, namely sustained tumor regression, complete local tumor eradication including microscopic brain infiltration, beyond a good “partial response,” as well as prevention of brain injury which presents unsightly and unpresentable radiographic change requiring unplanned intervention would therefore ultimately be necessary. In this regard, currently prevailing SRS schemes with extremely limited fractionation would have substantial limitations, given the far limited brain tolerance to ≤5 fr SRS, as previously described [3-5].

To overcome the limitations of ≤5 fr SRS, the appropriate implementation of >5 fr SRS would be expected and required, which has yet to be established. Since the first clinical introduction in February 2010, 10 fr SRS with forward-planned dynamic conformal arcs (DCA) has been adopted to BM cases deemed not amenable to ≤5 fr. These include those of >3 cm in diameter, in a critical location, and/or in the vicinity of vital structures [6-8]. Until 2017, 42 Gy, a BED_10_ of ≈60 Gy, with 70-80% isodose (the isocenter dose: 52.5-60 Gy, BED_10_ of 80-96 Gy), was usually prescribed to the PTV margin (GTV + isotropic 1 mm margin), in which the GTV marginal dose (e.g., D_98%_) was usually variable and inherently obscure [16]. In the DCA plans, the dose gradient outside the PTV (GTV + 1 mm) boundary was generally too steep to fully cover the inherent uncertainties, including inter- and intra-fractional changes and microscopic brain infiltration [15-17]. Through our eight-year experience, the authors concluded that the aforementioned dose (BED_10_) and its distribution were insufficient to achieve sustained tumor regression over six to 12 months at high rates, and both dose escalation and the remediation of dose distribution were necessary.

In 2013, Matsuyama et al. indicated that 2-10 fr SRS with a BED_10_ ≥80 Gy provided better local control for BM-derived from non-small cell lung cancer [18]. Their dose prescription was based on 90% isodose covering the D_95%_ of the PTV (GTV + 1-2 mm margin), a less inhomogeneous GTV dose [18]. They also referred to several cases of symptomatic brain necrosis that were refractory to medical treatment. This report was thus reframed, and a BED_10_-based dose prescription of ≥80 Gy was incorporated into our fSRS principles. To ensure further safety as well as improvement in local control, the authors therefore drastically modified their scheme as referred to previously, i.e., a BED_10_ of ≥80 Gy to “GTV” margin along with internal dose escalation and marked increment of fractionation. Since its clinical implementation in 2018, substantial improvement in the maximum response and higher progression-free rates at one year following fSRS have been observed for the majority of BM cases, as presented in this report. Notwithstanding this dose escalation, however, a few cases still represented pathologically verified or clinically unequivocal regrowth within less than two years. In our view, the GTV marginal dose of 53 Gy for 10 fr has thus far been acknowledged as a minimum requirement to attain long-term local control, especially in cases that do not receive anti-cancer medication. In the present parietal lesion, although part of the GTV margin eventually received <53 Gy, the planned GTV coverage with 53 Gy was 99.5%. If 53 Gy was rescaled to cover <98% of the GTV, complete tumor remission might not have been accomplished.

In addition to the optimum dose for tumor control, the dose-volume threshold accurately predicting the risk of radiation injury requiring unplanned treatment has remained unclear for 10 fr SRS. In that respect, the presented dose-volume relationships encompassing the irradiated isodose volumes in their entirety would be valuable for further broadening the analyses multi-directionally (Figure 4). The visible mass of 3.19 cm^3^ before lesionectomy probably included the necrosis derived from the brain parenchyma, and also the tumor itself, namely tumor necrosis. Even if this 3.19 cm^3^ mass consisted of only brain necrosis, the brain volume receiving ≥45 Gy would contribute predominantly to symptomatic brain necrosis, as the total volume receiving ≥48 Gy was 25 cm^3^ (>23.12 cm^3^ = 19.93 {initial GTV} + 3.19). Furthermore, this 3.19 cm^3^ volume was less than the decreased volume (7.71 cm^3^) of GTV during fSRS. For 5 fr SRS, the total volume receiving ≥20 Gy is associated with the risk of brain necrosis, while 20 Gy in 5 fr has generally been used as short-course WBRT [3-5]. Practically, higher dose volumes, such as ≥30 Gy, more precisely and circumstantially, the balance of both low- and high-dose volumes, would determine the severity of brain necrosis [19]. Furthermore, compared to the superficial location, the subcortical deep parenchyma, particularly the deep white matter adjacent to the lateral ventricle and/or corpus callosum, is intrinsically susceptible to radiation injury [20]. In the present case, unexpected higher dose exposure to the vulnerable paraventricular deep white matter was significantly related to a late detrimental effect. In general, an inhomogeneous target dose is linked to a steeper dose gradient outside the target, thereby minimizing the normal tissue dose and vice versa. Moreover, a steep dose increase just inside the GTV may be beneficial to conquer the potentially radioresistant internal hypovascular hypoxic condition and lead to early and noticeable tumor-shrinking during fSRT, but can also render the surrounding brain more susceptible to higher dose exposure. Thus, a very inhomogeneous GTV dose would be a double-edged sword that poses a dilemma and puts us in a quandary regarding the optimization of the degree of the dose gradient inside the GTV boundary.

The pattern of inter-fractional dorsomedial shifting in the presented parietal lesion bore a strong resemblance to that of the previously reported case, mainly solid type, in 2014 (case 3) [7]. These dynamic patterns may be predicted to some extent with respect to each location, histology (radiosensitivity), and/or features of affected brain deformities. Through further investigations with the accumulation of various inter-fractional changes, unusual and anomalous optimization of dose distribution, that is, intentional setting of a non-uniform anisotropic dose spillage margin outside the GTV and/or avoidance of steep dose increase inside the part of the GTV boundary could be considered on the assumption of the predictable implications for inter-fractional change [8].

In this case, the effect of a single dose of nivolumab (240 mg) on the clinical course, particularly the potential augmentation of anti-tumor efficacy, was not beyond the compass of speculation or imagination, including the possible abscopal effect. In any event, steroid administration for the relevant lung adverse reaction with tapering off over three months would substantially contribute to the stabilization of massive surrounding edema.

This report has several inherent limitations, and arguments based on this need further investigation. The two cystic lesions were not pathologically verified as SCC derived from the esophagus. Regarding the inter-fractional change, the potential tumor enlargement and/or shifting accompanied by increased edema during the two days awaiting fSRS cannot be denied, especially for the parietal lesion [9]. The follow-up period was limited to 2.5 years, so whether complete local tumor eradication is achieved in the brainstem lesion and whether the brainstem lesion represents symptomatic radiation injury hereafter has remained uncertain, along with whether the radiation injury in the parietal lobe tranquilizes hereafter.

In any case, the optimum dose distribution and candidate suitable for a 10 fr SRS dose should be determined through further investigations of a large number of cases on the assumption that fSRS entails the dynamic and unpredictable nature of the actual absorbed doses to both the tumor and surrounding brain during the period from planning image acquisition to irradiation completion.

## Conclusions

Ten fraction SRS with sufficient BED_10_ along with an appropriate dose gradient near the GTV boundary can provide superior tumor response and safety for BM not amenable to ≤5 fr SRS. A very inhomogeneous GTV dose can contribute to early and sufficient tumor shrinkage, symptom alleviation, and subsequent local tumor eradication, however, significant tumor shrinking and/or displacement during fSRS inevitably results in unnecessary higher dose exposure to the surrounding brain, which can lead to late radiation injury requiring intervention.
